# Impact of long-lasting insecticidal nets on prevalence of subclinical malaria among children in the presence of pyrethroid resistance in *Anopheles culicifacies* in Central India^☆^

**DOI:** 10.1016/j.ijid.2017.02.001

**Published:** 2017-04

**Authors:** Mehul Kumar Chourasia, Raghavendra Kamaraju, Immo Kleinschmidt, Rajendra M. Bhatt, Dipak Kumar Swain, Tessa Bellamy Knox, Neena Valecha

**Affiliations:** a National Institute of Malaria Research (ICMR) IIR-WHO Project, Field Unit, Kondagaon, Chhattisgarh, India; b National Institute of Malaria Research (ICMR), Sector-8, Dwarka, New Delhi 110077, India; c Department of Infectious Disease Epidemiology, London School of Hygiene and Tropical Medicine, London, UK; d National Institute of Malaria Research (ICMR), Field Unit, Lalpur, Raipur Chhattisgarh, India; e World Health Organization, Avenue Appia, 1211 Geneva-27, Switzerland

**Keywords:** LLIN, Subclinical malaria, Malaria, Pyrethroid resistance, India

## Abstract

**Background:**

Subclinical (asymptomatic) cases of malaria could be a major barrier to the success of malaria elimination programs. This study has evaluated the impact of long-lasting insecticidal nets (LLINs) on the prevalence of subclinical malaria in the presence of pyrethroid resistance in the main malaria vector *Anopheles culicifacies* on malaria transmission among a cohort of children in villages of the Keshkal sub-district in Chhattisgarh state.

**Methods:**

A cohort of 6582 children ages less than 14 years was enrolled from 80 study clusters. Post monsoon survey was carried out at baseline before LLIN distribution, and 5862 children were followed up in the subsequent year. Study outcomes included assessment of subclinical malarial infections and use of LLINs among the study cohort in the presence of varied levels of pyrethroid resistance.

**Findings:**

In the baseline survey, the proportion of subclinical malaria was 6·1%. LLIN use during the previous night was 94·8%. Overall, prevalence of subclinical malaria was significantly reduced to 1% (p < 0·001) in the second survey. LLIN users were protected from malaria (OR: 0·25, 95% CI = 0·12–0·52, p < 0.001) and subclinical malaria (OR: 0·25, 95% CI = 0·11–0·58, p = 0·001) despite the presence of pyrethroid resistance in the study area.

**Interpretation:**

In this low transmission area, sleeping under LLINs significantly reduced the burden of malaria among children. In the presence of pyrethroid resistant malaria vector, a high LLIN use of 94·5% was observed to have significantly brought down the proportion of subclinical malaria among the cohort children.

## Highlights

• Baseline prevalence of subclinical malaria infection was 6.1% with predominance of *P. falciparum* infections (67.6%).
• Subclinical malaria prevalence were significantly reduced by 84% in the follow up survey compared with baseline.

## Background

Vector control strategies including universal coverage of long-lasting insecticidal nets (LLINs) during the last decade and a half have significantly contributed to overall reduction in global malaria morbidity by 30%.^1^ In India, during the last decade, malaria morbidity has been reduced by 45% from an estimated two million reported malaria cases in year 2000 to 1·1 million cases in 2015 due to sustained control efforts.^2^ Of the remaining malaria cases, more than 80% are contributed by tribal forested areas of ten states including Chhattisgarh.^3^

Most malaria endemic countries, including India, have expanded their focus from malaria control to elimination, and India has recently launched a framework for a national level malaria elimination programme 2016–2030. This plan envisages scaling up of existing interventions, appropriate vector control measures, capacity building and strengthening drug compliance among malaria positive cases.^4^ At the time when the malaria control program is focused on targeting malaria vectors through indoor residual spraying (IRS) of insecticide and LLINs, there is a strong need to target malaria parasites and sub clinical carriers to interrupt malaria transmission in endemic areas.^5^, ^6^, ^7^

Under the existing health care system, malaria positive cases are captured through active and passive fever surveillance mechanisms. Persons with confirmed blood parasitemia through either microscopy or rapid diagnostic test (RDT) receive anti-malarial treatment. However, a sizeable proportion of the population has been observed to harbour malaria parasites without presentation of any symptom. Such cases are not captured through the routine surveillance system. As carriers, they pose a serious challenge to efforts towards malaria elimination.^8^, ^9^

Several studies from malaria endemic countries have reported that irrespective of the intensity of transmission, a large proportion of malarial infections remain asymptomatic and contribute about 20–50% of all malaria transmission.^10^, ^11^, ^12^, ^13^ At high levels of gametocyte parasitemia, subclinical carriers become the parasite reservoir^14^, ^15^ and contribute to persistent transmission of malaria.^5^, ^16^ Subclinical infections, also known as asymptomatic or sub-patent infections, have no standard definition, and different studies have used their own definition with subtle differences.^9^

In endemic areas asymptomatic parasitemia may confer partial immunity on repeated exposure and may provide protection against clinical manifestations.^17^, ^18^ Malaria parasitemia thus remains asymptomatic and acts as a source for residual transmission. However, the clinical consequences of subclinical malaria are not fully understood as they vary with different ecological, epidemiological and environmental conditions.^19^

There is a dearth of Indian studies evaluating impact of LLIN use on subclinical malarial infection and malaria transmission in pyrethroid resistant areas. Here, we present the results of a study on the impact of community-wide use of LLIN on the prevalence of subclinical malaria in a cohort of children under age 14 years in the presence of pyrethroid resistance in malaria vectors on the protective effectiveness of LLINs.

## Methods

The study was carried out during the post-monsoon seasons (August − December) of 2014 and 2015 among a cohort of children living in tribal forested villages in southern Chhattisgarh state, India.

### Study area

Keshkal (20° 5' 1 N and 81° 35' 12 E), a sub-district of Kondagaon district, has a population of about 90,000 living in 124 villages. A basic health care facility is available through one Community Health Centre (CHC) situated at block headquarter Keshkal, as well as four Primary Health Centres (PHCs), each catering to a population of about 20000–25000. Malaria transmission takes place mainly during the rainy season (June − October). *Anopheles culicifacies* is the principal vector in the region.^20^ For the past two decades, synthetic pyrethroid insecticide alpha-cypermethrin (@ 25 mg/m^2^ and twice a year) has been used in IRS as a major vector control measure by the state health department in the study area. With a consideration on year-round accessibility, 80 clusters were selected for the study. In 2014, 31,000 LLINs were distributed in collaboration with the state health department to cover a population of nearly 74,000 people living in these 80 clusters. In the remaining non-study clusters, the state health department has distributed LLINs covering nearly 16,000 population.

### Enrolment of children and baseline data collection

A cohort of 6582 children aged under 14 years was enrolled (60–80 children/cluster) after reading out the purpose of the study and obtaining written informed consent from the parent/guardian. Peripheral blood smear of all cohort children was prepared for microscopic confirmation of parasitemia to assess parasite prevalence before inclusion. Axial temperature of all study children at the time of survey was recorded with a digital thermometer (Dr. Diaz, Hemodiaz Life sciences Pvt. Ltd, India). Blood smears were stained with Geimsa stain and 100 microscopic fields of thick smear were examined at 1000 x magnification to detect malaria parasites. Parasite density (parasites/μl blood) was counted against 200 White Blood cells (WBCs) considering the average of 8000 WBC/μl. All slide positive malaria cases were treated with anti-malarial drugs according to national drug policy [*Plasmodium falciparum* (Pf): artesunate + sulfadoxine − pyrimethmine + primaquine; *P. vivax*(Pv):chloroquine + primaquine]. Follow up slides were prepared seven days after medication to ensure clearance of asexual parasites from the peripheral blood.

#### Case definition

A slide positive case (presence of asexual parasitemia) with no symptoms of fever (axial temperature ≥ 99·5 °F/37·5 °C) was considered as a case of subclinical malaria.

### Active case detection

For routine fever surveillance, 30 malaria surveillance workers were recruited from different clusters (villages) and trained in malaria surveillance and treatment activities. To assist them in malaria surveillance in houses of cohort children, 124 female Community Health Volunteers i.e. mitanins, were deployed. All cohort children were visited once in a fortnight. A follow up survey of all cohort children was carried out after six months of enrolment. Axial temperature was recorded using a digital thermometer. Self-reported history of fever during the preceding week, previous night and at the time of blood smear collection was recorded. Information on reported use of LLINs during the previous night was also recorded. However, in the follow up survey, ∼10% of children were lost to follow up due mainly to their exiting the study area to pursue higher studies elsewhere.

### Insecticide resistance assessment

In the year 2015, wild caught full-fed *An. Culicifacies* adult females caught from each cluster (∼100 females per test) were exposed to deltamethrin 0·05% treated papers following WHO test procedures to determine susceptibility status (WHO, 2013). Based on median mortality the clusters were stratified into low resistance (≥84% mortality) and high resistance (<84% mortality) clusters. Thus, 3,249 and 3,333 cohort children were allocated to low resistance and high resistance clusters respectively.

### Statistical analysis

Data were entered in EpiData version 3·1 software. All entries were double checked and further cleaned for error and analysed using IBM SPSS version 20 (IBM Corp, Armonk, NY). Continuous variables were described as mean and standard deviation (M ± SD), and categorical variables were described in percentages.

To assess the reduction in prevalence of malaria and subclinical malaria cases in consecutive years, the data for both surveys were combined into one data set and included ‘survey year’ as a fixed effect in the generalised estimated equation (GEE) model together with all the other variables. Reduction in malaria from one survey to the next, adjusted for all the other variables in the model were analyzed.

Effect of LLIN use between cluster resistance status was determined in a separate stratified analysis, and then overall cross level interaction between the two was analyzed by adding interaction term in the GEE model to assess impact of insecticide resistance on LLIN effectiveness.

Univariate and multivariate analyses were carried out using a generalised estimated equation (GEE) regression model to explore the relation between subclinical malaria and exposure variables. GEE analysis was chosen so that within-cluster correlation can be adjusted and possible interaction of village resistance status can be taken into account during modelling. Response variable presence of subclinical malaria has binary events (Yes/No). Binomial distribution with logit link function was selected. Exchangeable correlation structure was chosen for the model. In the model, explanatory variables that included age group, gender, and last night LLIN use were added and village insecticide status was taken as main effect. Study cluster variable was taken at the subject level. The results of GEE regression analyses were stated as unadjusted and adjusted odds ratio (OR) with 95% confidence interval (CI) and associated *P*-value.

### Informed consent and ethical clearance

The purpose of the study was described in the local language to all study participants, and study related queries such as routine surveillance and blood slide collection procedures etc. were addressed. A written informed consent was collected from the parents or guardians of the enrolled children. This study was undertaken as a part of a WHO-coordinated multi-country project and ethical clearance was obtained from the Institutional Ethics Committee (ECR/NIMR/EC/2010/75).

## Results

### Demographic and clinical characteristics

Between September and November 2014, 6582 children (Male: Female − 1:1·03) were enrolled from 80 study clusters. The mean age of the study cohort was 6·3 years (SD- 3·4); children of age group 5–9 years constituted 40% of the study population followed by 2–4 years (28·9%). Of all children, 114(1·7%) and 196 (3·3%) reported with a history of fever (i.e., the previous night or at the time of survey) in the baseline and follow up surveys respectively. Detailed demographic and clinical description of the study population is given in Table 1.

In the baseline survey, peripheral blood samples from a total of 490/6582 (7·4%) children had malarial parasites (Table 2). A total of 398 (81·2%) microscopy positive children presenting without any symptoms at the time of survey had either *Pf* (331) or *Pv* (67) parasitemia. One child had a subclinical infection with *P. malariae.* In all, 91 microscopy positive children (1·4%) had malarial infection (*Pf* = 87; *Pv* = 4) associated with clinical symptoms. Among *Pf* infections, asexual parasitemia was higher (GMD 1680 parasites/μl, range 40–508880) in symptomatic cases as compared to asymptomatic cases (GMD 717 parasites/μl, range 40–46760). Similarly, sexual (gametocyte) parasitemia in symptomatic *Pf* infections was nearly two times higher than in asymptomatic children (GMD 613 vs 303 parasite/μl).

In the follow up survey carried out during 2015, blood samples of 5862 children were collected, of which 82 (1·4%) were positive for malaria parasites. Among the positives, 46 (56·1%) and 19 (23·2%) children had subclinical *Pf* and *Pv* infections, respectively.

### Comparative analysis of subclinical malaria and total malaria cases in baseline and follow up survey

The prevalence of malaria was significantly reduced during the follow-up survey in August 2015 as compared to the baseline survey in November 2014 (SPR = 7·4% vs 1·4%, p < 0·001) after adjusting other factors such as gender and age groups. Similarly, significant reduction was observed in the prevalence of subclinical malaria in children in baseline and follow up surveys (SPR = 6·1% vs. 1%, p < 0·001) keeping other variables (gender and age group) constant. (Table 3).

### Effect of insecticide resistance on protective efficacy of LLIN

Mean (range) mortality rate of *An. culicifacies* to pyrethroid in high and low resistance clusters was 71·8% (53·0–83·6) and 93·8% (84–100) respectively. Malaria positive cases both clinical and subclinical were stratified based on susceptibility test data of *An. culicifacies* to deltamethrin of year 2015. Clusters with high deltamethrin resistance *An. culicifacies* showed almost equal numbers of subclinical malaria cases compared to low resistance clusters (1% vs. 1·4%). Overall, no interaction between the LLIN uses and insecticide resistance of study clusters was observed (Adjusted OR,95%CI- 0.58, 0.23-1.4; p = 0.21). (Table 4).

### Impact of LLIN uses and other explanatory factors associated with subclinical malaria

Table 5 shows results of the GEE univariate and multivariable analysis, which demonstrated that last night use or non-use of LLIN was one of the significant independently associated factors (OR = 0·25, 95% CI = 0·11, 0·58, p = 0·001), with non-use associated with higher prevalence of subclinical malaria. However, malarial infection frequency was nearly similar in both the age groups, 0–4 and 5–14 years (0·9% vs. 1·2%, p = 0·297) and between genders (1·2% vs. 1%, p = 0·811) with no significant association with subclinical malaria identified.

## Discussion

Identification and targeting of subclinical malaria cases is one of the key challenges for countries aiming for malaria elimination.^9^ Children under the age of five years and between 5–14 years old are most vulnerable to malaria infections. Malaria prevalence among these age groups and LLIN usage the previous night are among the few key indicators used to measure outcomes of malaria intervention programs.^21^ However, asymptomatic malaria cases have also been noted as a suitable indicator to assess the success of programs.^22^ Systematic reviews have clearly suggested that LLINs, a definitive component of malaria control programs in most settings, if used properly, have significant impact in reducing malaria incidence in endemic communities.^23^, ^24^ However, the impact of LLIN use on subclinical malarial infections and residual transmission has largely remained unknown.

The findings of the baseline survey (2014) showed that overall prevalence of subclinical malaria infection was 6·1%, constituting more than three fourths of total slide positive cases (81·3%) among the enrolled children with predominance of *P. falciparum* infections (67·6%). Studies from different parts of the world have reported that subclinical malaria burden could be 4–5 times of total reported malaria cases,^10^ even in low transmission areas.^25^ In India, various sporadic cross-sectional studies have suggested that asymptomatic parasitemia prevalence could range from 2·9% to 8·4% in the transmission season.^8^, ^26^, ^27^, ^28^ Thus, it can further be speculated that a substantial proportion of subclinical malarial infection may exist, which routinely remains undetected in the existing surveillance system.

An active case surveillance (ACS) system to monitor the fever cases and to ensure regular LLIN use in the selected houses showed that LLIN use among cohort children (under 14 years old) in the study area was 94·8%, which was comparatively higher than reported in other studies.^29^ This higher LLIN use can be attributed to regular fortnightly surveillance and concurrent health education by field staff during their visits. Another plausible explanation is that regular visits of health workers led to positive response bias among the respondents. However, successive attempts were made to crosscheck the proxy indicators (hanging practices of bed nets) as well as house-to-house health education and awareness campaigns to improve LLIN usage^30^ since knowledge about malaria transmission and educational level of head of the household is also an important predictive factors for LLIN utilization.^29^

From this study, it can be stated that use of LLINs was equally effective in protecting the cohort from clinical malaria and subclinical malarial infections. A possible hypothesis for the protective effect of LLIN against subclinical infection is that there was interruption of transmission at the household level. It has been observed that if malaria infection were diagnosed from a member of one household there would be increased chance of presence of subclinical infection in other members of the family.^31^ Secondly, acquired immunity among the children as well as adults^11^ can also be a plausible reason that these asymptomatic carriers can act as source of infection at household level.

Malaria incidence and subclinical malaria prevalence were significantly reduced by 84% in the follow up survey relative to the baseline, which was perhaps attributable to adequate LLIN coverage and high usage rates by the cohort children. Nonetheless, ACS also substantially contributed to the control of malarial infection in the study population. A study from Kenya showed a similar decrease in subclinical malaria due to regular ACS.^32^ In the Asia Pacific region, different countries have implemented variants of active case surveillance systems to identify and reduce parasitic carriers among reservoirs. Case detection and reactive active case detection were among the approaches taken, although better and more effective methods are still required.^33^

Parasite density, age and season are essential co-variates in risk of developing fever.^34^ However, on the contrary, no major difference in age and parasite density among subclinical and total malaria cases was observed in the present study. Asymptomatic parasitemia is usually higher in the dry season as compared to the rainy season^35^ and the presence of gametocyte density in the dry season probably acts as a source of infection in the next cycle of malaria transmission.^36^ In the Keshkal sub district, malaria transmission is seasonal and malaria incidence usually peaks in the post monsoon season. It has also been reported that increased breeding potential and subsequently vector density during monsoon and post-monsoon seasons directly affect the malaria transmission in low and meso-endemic regions.^29^, ^34^ In view of this, to control for this confounding effect due to seasonal variation, both the surveys were conducted in the same season in the consecutive years of 2014 and 2015. However, there might be a chance of year-to-year variation due to climatic factors.

The worldwide spread of insecticide resistance among malaria vectors has been well-established.^21^ It was assumed that pyrethroid resistance can adversely affect efforts of malaria control and elimination, though no concrete evidence is yet available although there have been a few studies^37^, ^38^ that suggest an association between insecticide resistance and failure of malaria interventions. However, under laboratory and hut trial conditions it has been shown that LLIN remains effective despite pyrethroid resistance in main malaria vector species.^39^ In India, occurrence of pyrethroid resistance has been reported in malaria endemic states including Chhattisgarh.^20^, ^40^

Susceptibility tests carried out in 2015 have confirmed pyrethroid resistance in *An. Culicifacies* in most of the study clusters (69) based on WHO cut off ≥98% of mortality rate. Stratified data analysis showed that the occurrence of malaria infection among LLIN users and non-users was similar and resistance status was not behaving as an effect modifier for LLIN usage and malaria cases. We speculate that this could be due to higher LLIN usage among the children and no loss of effectiveness in LLIN efficacy in protecting from malaria and subclinical malaria was observed.

Apart from seasonal dynamics of malaria transmission, residing in malaria hotspots at micro-geographical scale in low transmission areas is an independent predictive factor for malaria infections.^41^ Cluster wise data of both the surveys identified few independent clusters with comparatively higher number of malaria cases, which might be due to their proximity to breeding habitats. Such clusters may serve as a potential reservoir of subclinical cases. Targeting these subsets of the population with appropriate interventions could be cost-effective in clearing the residual parasite reservoir.^42^, ^43^ However, this needs to be further corroborated by geospatial assessment, and entomological studies.

While this study has focused on the post-monsoon season, further assessment is required during the dry season, when LLIN utilization might reduce due to reduction in vector densities or hot climates that deters usage. Further follow up could delineate the effect in prevalence of subclinical malaria in the low transmission season as well.

This study presents an ideal scenario with high LLIN use, and well-informed household and study findings could only be generalized to other tribal populated area within low transmission settings, if high LLIN coverage is maintained with a robust active surveillance activity in place.

## Conclusions

There was a high proportion of subclinical malaria infections among children in the tribal inhabited malaria endemic area of the study, which may also be the case in areas with similar populations and malaria transmission intensities, and therefore may inform appropriate malaria elimination strategies. These observations indicated that high use of LLINs can significantly reduce the number of subclinical malaria cases, despite vectors having reduced susceptibility to the pyrethroid used in the LLINs. Therefore, high LLIN coverage and usage supported by a strong active case surveillance system would be a useful strategy in low transmission settings where parasite reservoirs exist as subclinical carriers.

## Conflict of Interest

No competing interest declared

## Role of funding source

Funding was provided by the Bill and Melinda Gates Foundation (Grant number OPP 1062754) which has no role in the planning, study design, data collection or write up. This research forms part of a multi-country study coordinated by the Global Malaria Programme of the World Health Organization.

## Ethics approval and consent to participate

The purpose of the study was described in the local language to all study participants, study related queries such as routine surveillance, and blood slide collection procedures etc were addressed. A written informed consent was collected from the parents or guardians of the enrolled children. Ethical clearance was obtained from the Institutional Ethics Committee (ECR/NIMR/EC/2010/75).

## Figures and Tables

**Table 1:** Demographic and clinical characteristics of study population (n = 6582).

**Variable**	**Category**	**Percent (n)**	
**Age (in years, 2014)**	Mean (SD)	6·3 (3·4)	
	<2	7·3 (480)	
**Age group (in years, 2014)**	2–4	28·9 (1899)	
	5–9	40·0 (2634)	
	10–14 Years	23·8 (1569)	
**Gender**	Male	49·3 (3246)	
**Insecticide resistance status (Deltamethrin 0·05%, 2015)** **Median mortality- 84%**	Low resistance clusters (Bioassay mortality ≥84%)(40)	50·6 (3333)	
	High resistance clusters (Bioassay mortality <84%) (40)	48·7 (3249)	
		**Baseline 2014** **(n** **=** **6582)**	**Follow up 2015** **(n** **=** **5862)**
**Slide positivity rate**	Microscopy positive	7·4 (490)	1·4 (82)
**Clinical malaria (with fever)**	Yes	1·4 (91)	0·26 (17)
**Subclinical Malaria** **(without fever)**	Yes	6·1 (399)	1 (65)
**Previous night fever history**	Yes	1·7 (114)	3·3 (196)
**Loss to follow up**		–	10·9 (720/6582)

**Table 2:** Host and parasite characteristics of study cohort during inclusion and follow-up.

**Variable**	Asymptomatic		Symptomatic		Total malaria positive
	*P. falciparum*	*P. vivax*	*P. falciparum*	*P. vivax*	
**Baseline survey (n** **=** **490 positives/6582 slides; SPR** **=** **7·4%)**					
**Number (%)**	331 (67·6)	67 (13·7)	87 (17·8)	04 (0·8)	490#
**Age, Mean** **(years; range)**	6·6 (1–12)	6·5 (1–12)	6·6 (1–12)	6·7 (4–11)	6·6 (1–12)
**Males, n (%)**	159 (48·0)	36 (53·7)	41 (47·1)	2 (50)	238 (48·6)
**Asexual parasite density,** **geometric mean** **(per μl, range)**	717	415	1680	247	
	(40–46760)	(40–36880)	(40–508880)	(80–760)	
**Sexual parasite density,** **geometric mean** **(per μl, range)**	99	303	157	613	
	(40–1720)	(40–49040)	(40–1440)	(40–31880)	

**Follow up survey (n** **=** **82 positives/5862 slides; SPR** **=** **1·4%)**					
**Number (%)**	46 (56·1)	19 (23·2)	14 (17·1)	03 (3·7)	82 (100)
**Age, Mean** **(years; range)**	7	6	7	3	7
	(1–12)	(1–12)	(3–12)	(2–4)	(1–12)
**Males, n (%)**	21 (45·7)	12 (63·2)	05 (26·3)	01 (33·3)	39 (47·5)
**Asexual parasite density,** **geometric mean** **(μl, range)**	892	164	8402	1560	
	(40–340400)	(40–1480)	(80–628440)	(1560)	
**Sexual parasite density,** **geometric mean** **(μl, range)**	86	320	Nil	1037	
	(40–440)	(40–8200)		(360–5160)	

# One case of *P*. *Malariae* was also identified and is included in the total malaria cases.

**Table 3:** Comparison of subclinical malaria cases in baseline and follow up survey (n = 12444).

Parameters	Category	Positive (%)	Total (N = 12444)	Unadjusted Odds ratio (95%CI)	P value	Adjusted OR (95%CI)	P value
Survey Year	2015	65(1)	5862	0·196 (0·18–0·26)	<0·001	0·194 (0·15–0·26)	<0·001
	2014	399(6·1)	6582				
Gender	Male	236(3·7)	6111	1·06 (0·86–1·31)	0·594	1·06 (0·85–1·32)	0·631
	Female	228(3·7)	6333				
Age groups	5–14 years	325(3·8)	8479	1·04 (0·87–1·3)	0·661	1·20 (0·98–1·54)	0·07
	0–4 years	139(3·5)	3965				
Subject level- study cluster (n = 80)							

**Comparison of malaria cases in baseline and follow up survey(n** **=** **12444)**							
Survey Year	2015	490(7·4)	5862	0·203 (0·15–0·27)	<0·001	0·20 (0·15–0·27)	<0·001
	2014	82(1·4)	6582				
Gender	Male	277(4·5)	6111	1·02 (0·86–1·21)	0·810	1·02 (0·85–1·22)	0·87
	Female	295(4·7)	6333				
Age groups	5–14 years	396(4·7)	8479	1·01 (0·86–1·2)	0·915	1·2 (0·97–1·38)	0·11
	0–4 years	176(4·4)	3965				
Subject level- study cluster(n = 80)							

**Table 4:** Malaria cases stratified according to insecticide resistance status in *An. Culicifacies* (n = 5862).

**Last night LLIN usage vs. malaria in 2015**							
Insecticide resistance status	Variable	Category	Microscopy Positive 2015		Stratified Odds ratio (OR), 95%CI	P value	Adjusted OR 95%CI, p value
			(%)	Total			
Low resistance	Last night LLIN use	Yes	41 (1·4)	2840	0·30, 0·09–1·00	0·05	0·58
		No	05 (4·7)	107			0·23–1·4
		Total	46 (1·6)	2947			0·21
High resistance	Last night LLIN use	Yes	27 (1)	2718	0·21, 0·08–0·55	**0·001**	
		No	09 (4·6)	197			
		Total	36 (1·23)	2915			

**Last night LLIN usage vs. Subclinical malaria in 2015**							
			Subclinical malaria 2015				
Low resistance	Last night LLIN use	Yes	33 (1·2%)	2840	0·30, 0·08–1·1	0·07	0·56
		No	04 (3·7%)	107			0·21–1·4,
		Total	37 (1·26%)	2947			0·22
High resistance	Last night LLIN use	Yes	21 (0·8%)	2718	0·21, 0·07–0·66	**0·008**	
		No	07 (3·6%)	197			
		Total	28 (1%)	2915			

**Table 5:** Prevalence of subclinical malaria by use of LLINs, gender, age-group and insecticide resistance status of study clusters (n = 5862).

Parameters	Category	Positive (%)	Total (N = 5862)	Unadjusted Odds ratio(95%CI)	P value	Adjusted OR (95%CI)	P value
Last night LLIN use	Yes	54(1)	5558	0·26 (0·11–0·60)	0·002	0·25 (0·11–0·58)	0·001
	No	11(3·6)	304				
Gender	Male	33(1·2)	2865	1·08 (0·6–1·95)	0·799	1·08 (0·6–1·94)	0·811
	Female	32(1·1)	2997				
Age groups	5–14 years	46(1·2)	3749	1·40 (0·67–2·73)	0·394	1·33 (0·66–2·65)	0·297
	0–4 years	19(0·9)	2113				
Cluster resistance status	High	28(1)	2915	0·76 (0·30–1·98)	0·577	0·70 (0·27–1·81)	0·457
	Low	37(1·3)	2947				
Subject level- study cluster (n = 80)							

**Overall prevalence of infection by use of LLINs, gender, age-group and insecticide resistance status of study clusters (n** **=** **5862)**							
Last night LLIN use	Yes	68(1·2)	5558	0·26 (0·12–0·54)	<0·001	0·25 (0·12–0·52)	<0·001
	No	14(4·6)	304				
Gender	Male	39(1·4)	2865	0·95 (0·56–1·59)	0·840	0·94 (0·56–1·59)	0·825
	Female	43(1·4)	2997				
Age groups	5–14 years	64(1·5)	3749	1·32 (0·74–2·38)	0·350	1·29 (0·73–2·29)	0·382
	0–4 years	18(1·1)	2113				
Clusters resistance status	High	36(1·2)	2915	0·80 (0·30–2·07)	0·629	0·72 (0·28–1·88)	0·504
	Low	46(1·6)	2947				
Subject level- study cluster (n = 80)							
